# Mobile Admission Process and Administrative Turnaround Time for Hospitalization of Outpatients: A Retrospective Study

**DOI:** 10.1055/a-2576-7110

**Published:** 2025-08-14

**Authors:** Ho Sub Chung, Myeong Namgung, Sung Jin Bae, Yunhyung Choi, Dong Hoon Lee, Chan Woong Kim, Sunho Kim, Kwang Yul Jung

**Affiliations:** 1 Department of Emergency Medicine, College of Medicine, Chung-Ang University Gwangmyeong Hospital, Gwangmyeong-si, Republic of Korea

**Keywords:** hospital administration, patient admission, telemedicine, workflow

## Abstract

**Objectives:**

This study compared the time efficiency of the hospital admission process using personal mobile devices to traditional walk-in methods, thereby assessing the effectiveness of the mobile admission process.

**Methods:**

This retrospective study was conducted at Chung-Ang University Gwangmyeong Hospital in South Korea (August 2022–January 2023). Turnaround times for the walk-in and mobile admission processes were compared. Patients were divided into mobile and walk-in groups based on their admission process. Collected timestamp data were extracted by examining patients' electronic medical record log time or caregivers' electronic signatures on consent forms. Time intervals between timestamp data were calculated and compared.

**Results:**

We enrolled 4,344 patients to compare the turnaround time and demographics of the mobile ( *n* = 1,336) and walk-in ( *n* = 3,008) admission processes. The former had a significantly shorter mean turnaround time (13.4 minutes) than the latter (22.2 minutes). Female patients, younger patients, and those admitted to surgery departments were more likely to use the mobile process. Older patients were less likely to undergo mobile admissions. A linear regression analysis revealed that these factors significantly affected the usability of the mobile device admission process.

**Conclusion:**

Compared with the traditional walk-in admission process, the mobile admission process can reduce task completion time.

## Background and Significance

Hospitals aim to enhance patient care quality while reducing costs and increasing revenue. Consequently, concepts such as process optimization, throughput, and efficiency are considered essential for achieving operational objectives in healthcare. 1 Additionally, hospitals have been exploring methods to digitize their processes for improving patient experience and optimizing operations. 2 3 The medical field has been focusing on digital transformation to improve workflow. 4 5

However, administrative tasks, such as the hospital admission process, involving patients and hospital staff, are handled inefficiently. Traditionally, patients undergo the time-consuming process of completing paperwork and waiting in line before admission. The admission process entails verifying a patient's identity and having necessary documents signed for hospitalization; patients experience inefficiency and dissatisfaction because of the long waiting time and confusing process flow.

Lately, several hospital tasks have been managed using cell phones. 6 Mobile devices' portability and ubiquity allow users to function efficiently. 7 Mobile device use in hospitals for diagnosing and improving treatment efficacy based on personal health records 8 9 10 and mobile electronic medical records' adoption for workflow improvement by medical staff are actively being researched. 11 12 13 However, research on the effects of utilizing these technologies for administrative task improvement is scarce. Improving hospital administrative processes is crucial for enhancing work efficiency and patient satisfaction.

## Objectives

We used mobile applications to facilitate the hospital admission process, and determine their efficiency compared with the traditional in-person visit process.

## Methods

### Study Setting

This retrospective study was conducted at Chung-Ang University Gwangmyeong Hospital (692 beds) in Gyeonggi-do, South Korea. As of March 2023, it operated 380 patient rooms, including 23 intensive care units. Chung-Ang University Gwangmyeong Hospital maintains a Clinical Data Warehouse (CDW) approved by the Institutional Review Board (IRB). Data obtained from the CDW are de-identified and processed to ensure patient anonymity and confidentiality. The data used in this study were extracted from the CDW. The study was reviewed and approved by Chung-Ang University Gwangmyeong Hospital's IRB (approval number CAUGH 2304–077–041), satisfying the requirements specified in the Ministerial Decree of Health and Welfare passed by the National Bioethics Committee. All methods were performed as per the relevant guidelines and regulations.

### Hospital Admission Process

In most hospitals in South Korea, patients arriving on their scheduled admission date typically proceed to the administrative department for identification and to complete various consent forms, such as admission-related agreements with the assistance of hospital administrative staff. The specific types of consent forms may vary by institution. Subsequently, they proceed to the admission guide booth to provide their clinical information, receive information about hospital life and safety, and sign the “Hospitalization Guide” form as an acknowledgment of understanding. Then, they proceed to their assigned wards; the nurse confirms their arrival and records the timestamp data.

**Fig. 1:**
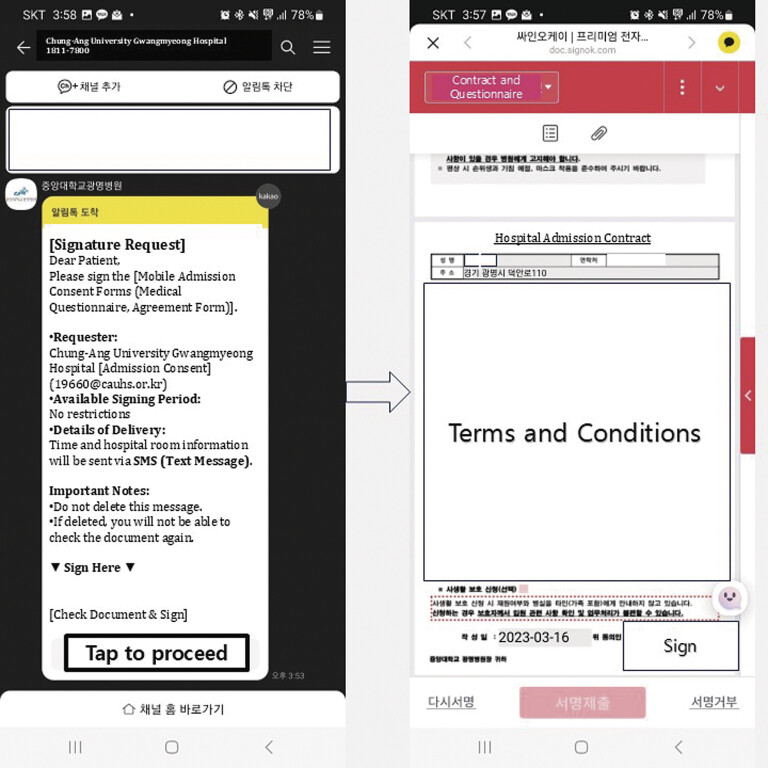
Captured screens of hospital admission process.

The hospital launched a mobile admission system on August 1, 2022, allowing hospital administrative procedures to be performed using the patient's mobile phone on the morning of admission date. All patients or caregivers are informed beforehand about the mobile admission process and given hospitalization instructions. The hospital's administrative department sends patients the “Admission Contract” form and consent forms via short message service (SMS) or mobile messenger applications, such as Kakao Talk, 24 hours before admission ( Fig. 1 ). Patients who submit the forms need not visit the administrative department; they can proceed directly to the admission guide booth.

### Study Participants

**Fig. 2:**
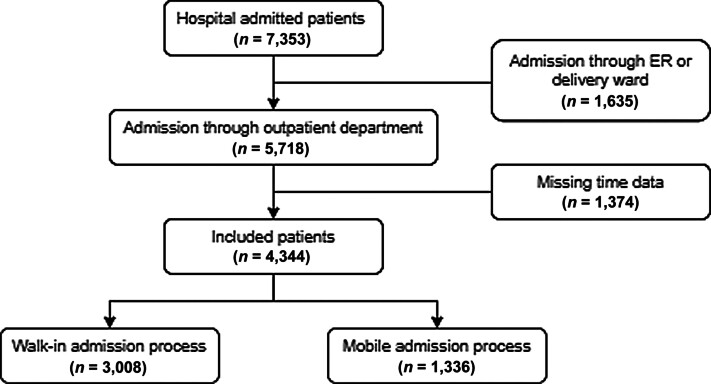
Flow diagram of the enrolled patients.

Between August 2022 and January 2023, 7,353 patients were admitted to the hospital. Fig. 2 depicts the participants' selection process. Following the inclusion and exclusion criteria, 4,344 patients were included in the analysis (3,008 walk-ins and 1,336 mobile admissions).

Patients admitted on weekdays with a doctor's admission prescription from the outpatient department were included. Those lacking a timestamp due to missing data resulting from an abnormal flow, such as patients not visiting the administration department or the absence of consent forms due to repeated admissions, such as for chemotherapy treatments, were excluded. Additionally, emergency room (ER) admissions were excluded as the admission process and transition within the ER differ significantly from the outpatient flow. Patients who underwent the admission process on weekends were excluded because the weekend flow differs from that of weekdays. We sorted patients into two age groups: older adults (≥65 years) and adults (<65 years). Pediatric patients were included in the adult group because adult caregivers accompanied them during hospital admission. Admission departments were categorized according to whether surgery was performed. The surgery section comprises general surgery, neurosurgery, thoracic surgery, otolaryngology, ophthalmology, obstetrics/gynecology, orthopedic surgery, plastic surgery, dentistry, and urology departments. The remaining cases were categorized as nonsurgical.

### Data Collection

**Fig. 3:**
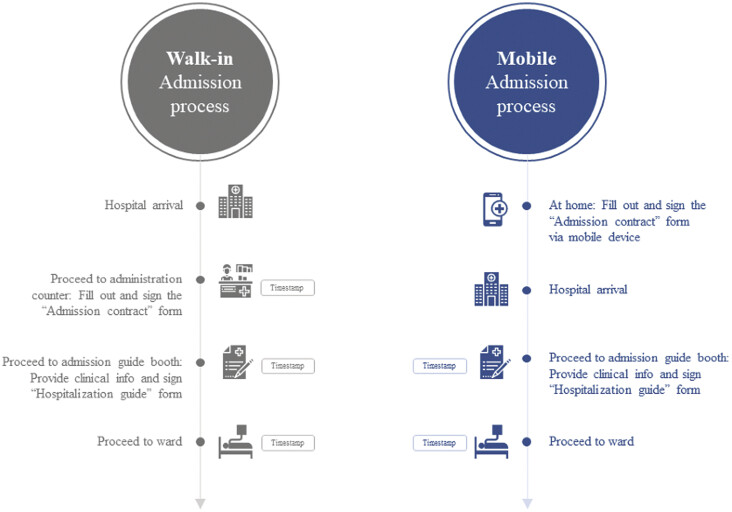
Comparison of the walk-in and mobile admission processes.

Chung-Ang University Gwangmyeong Hospital employs electronic signatures using tablets or other devices for all consent forms, which were integrated into the electronic medical record system. Examining the overall turnaround time of the admission process, from the patient's arrival at the hospital to the patient's arrival at the ward, required timestamp data for each step of the process. Hospital timestamp data were extracted and analyzed based on the flow of patients or caregivers during the admission process. Timestamps were extracted from electronic medical record log data when (1) patients or caregivers signed the “Admission Contract” form at the administration counter, (2) patients or caregivers signed the “Hospitalization Guide” form at the admission guide booth, and (3) patients arrive at the ward, as recorded by nurses. The time intervals between the timestamps were calculated. Fig. 3 summarizes the walk-in and mobile admission processes.

### Outcome Measures and In-depth Usability Analysis

The primary outcome was the turnaround time for each admission method. The factors associated with the mobile admission process' usability sorted by sex, age (adults and older adults), and admission department (surgery and non-surgery) were analyzed.

### Statistical Analysis

Continuous variables are presented as averages and standard deviations with 95% confidence intervals (CIs); categorical variables are presented as frequencies and percentages. The frequency difference between the two groups was examined by conducting Pearson's χ ^2^ analysis and Fisher's exact test. The average difference was examined by conducting Student's *t* -test. Statistical significance was set at *p* < 0.05. Each factor's influence on the time interval was investigated through a linear regression analysis. The relationship between the time interval and each factor was assessed through a univariate analysis, followed by a multiple linear regression analysis to obtain the adjusted time interval. R version 4.2.0 (2022–04–22; R Foundation, Vienna, Austria) was used for all statistical analyses.

## Results

### Main Outcome

Younger and female patients used the mobile admission process more often than others. The total turnaround time for the admission process was significantly shorter for the mobile (median: 13.4 minutes) than for the walk-in process (median: 22.2 minutes; Table 1 ).

**Table 1:** Comparison of demographics and turnaround time between the admission processes

	Category	Overall	Mobile	Walk-in	*P* value
*n*		4,344	1,336	3,008	
Sex = male (%)		1,605 (36.9)	356 (26.6)	1,249 (41.5)	<0.001
Age (mean [SD])		55.1 (16.9)	46.7 (12.8)	58.86 (17.2)	<0.001
Older adults (%)		1,304 (30.0)	71 (5.3)	1,233 (41.0)	<0.001
Department = surgery (%)		2,890 (66.5)	1,009 (75.5)	1,881 (62.5)	<0.001
Admission month (%)					<0.001
	August	738	137 (10.3)	601 (20.0)	
	September	680	156 (11.7)	524 (17.4)	
	October	680	198 (14.8)	482 (16.0)	
	November	752	273 (20.4)	479 (15.9)	
	December	751	264 (19.8)	487 (16.2)	
	January	743	308 (23.1)	435 (14.5)	
Admission date (%)					<0.001
	Monday	947	271 (20.3)	676 (22.5)	
	Tuesday	934	270 (20.2)	664 (22.1)	
	Wednesday	994	372 (27.8)	622 (20.7)	
	Thursday	878	281 (21.0)	597 (19.8)	
	Friday	591	142 (10.6)	449 (14.9)	
Total min (median [IQR])			13.4 (17.7)	22.2 (30.4)	<0.001

Abbreviations: IQR, interquartile range; SD, standard deviation.

The relationship between sex, age, department, and time was assessed through a covariate analysis, adjusting the dependent variable values using the covariates. The time variable was adjusted by conducting a covariate analysis with sex, age, and department as the covariates.

### Factor Affecting the Time Interval Difference

Adjusted values for time were obtained through a multiple linear regression analysis, with sex, age, and department as independent variables and time as the dependent variable. The results showed that the mobile admission process took less time, consistent with the findings from the unadjusted time interval ( Table 2 ).

**Table 2:** Multiple linear regression analysis of the study outcomes

	Mobile				Walk-in			
	Estimate	SE	*t* Value	*p* -Value	Estimate	SE	*t* Value	*p* -Value
(Intercept)	31.7	4.0	8.0	<0.05	46.3	2.6	17.9	<0.05
Sex	−11.0	3.8	−2.9	<0.05	−4.8	2.5	−1.9	0.05
Age	4.6	7.3	0.6	0.53	0.1	2.4	0.1	0.96
Department	5.4	3.9	1.4	0.16	5.2	2.5	2.1	0.04
Unadjusted time interval, median (IQR)	13.4 (17.7)				22.2 (30.4)			
Adjusted time interval, median (IQR)	12.2 a (12.8)				22.2 b (30.4)			

Abbreviations: IQR, interquartile range; SE, standard error.

a Adjusted for sex.

b Adjusted for department.

### Factors Affecting Mobile Admission Process Use

Linear regression helped identify the factors associated with mobile admission process use, with all identified factors showing a significant effect. Female and surgery patients used the mobile admission process significantly more frequently than older adults ( Table 3 ).

**Table 3:** Linear regression results of the mobile admission process use

	Estimate	SE	*t* Value	Pr(>|t|)
(Intercept)	0.32	0.01	22.08	<0.05
Female	0.08	0.01	5.80	<0.05
Elderly	−0.34	0.01	−23.79	<0.05
Surgery	0.06	0.01	4.23	<0.05

Abbreviation: SE, standard error.

Note: Residual standard error: 0.4277.

## Discussion

### Principal Results

To our knowledge, this is the first study to use real-world data to assess mobile devices' usability in the hospital admission process and examine potential improvements. Time efficiency improved with the mobile admission process; the admission time reduced by approximately 9 minutes compared with the walk-in process. Surgery, female, and younger patients used the mobile admission process more than others.

Chung-Ang University Gwangmyeong Hospital, where the mobile admission process is continuously utilized, conducted a System Usability Scale (SUS)-based survey to assess its usability. Participation in this survey was voluntary and open to users who had experienced the mobile admission process. Over the course of 1 month, 40 patients or caregivers participated, yielding an average SUS score of 74.4; according to the SUS theory, this indicates good usability ( Supplementary Appendix 1 , available in the online version). A good usability score on the SUS reflects an intuitive and efficient system that enables users to achieve their goals with minimal learning effort and errors while providing a satisfying user experience. 14 Thus, consistent with the findings of this study, the mobile admission process not only enhances efficiency but also demonstrates high usability. However, to further validate these results, a more structured and large-scale study should be conducted to comprehensively assess its usability.

Overall, the mobile admission process allowed patients to complete the necessary paperwork from their homes, reduced waiting times, streamlined the admission process, and reduced the administrative staff's burden.

### Technical and Legal Concerns

The mobile admission process utilized existing and widely used mobile messenger applications and SMS for notification purposes. The administrative team directly handled patient inquiries regarding technical issues, which were minimal compared with the overall usage volume, indicating that the system's implementation was not excessively difficult. Moreover, patients can use the conventional walk-in admission process if the mobile process poses challenges.

Storing data and transmitting documents containing personal patient information via mobile devices is a sensitive issue. However, authentication methods employed by government agencies and financial institutions helped mitigate potential legal issues associated with accessing and verifying such documents.

### Improving Administrative Processes in Hospitals

Hospitals are working toward integrating new technologies in healthcare to achieve efficiency and cost reduction in administrative work. These technologies were increasingly applied during COVID-19, with more tasks performed without face-to-face interactions.

Previous studies have identified workflow improvements using mobile devices in clinical or medication-related areas within hospitals. 13 15 Administrative process improvements are often considered purely managerial issues not directly related to patients. However, this study highlights that administrative procedures involving patients can be effectively streamlined through the use of mobile devices, with the added significance of quantitatively validating this improvement.

Although eliminating unnecessary document-related tasks and conducting business remotely is ideal, hospitals' increasing legal liabilities require various consent forms. 16 17 18 This study confirmed that optimizing patient administrative tasks using mobile devices is the easiest way to utilize existing technologies. Moreover, expanding the scope of tasks using mobile devices improves hospital workflow and patient satisfaction. 19 Future research with a more structured design is warranted to evaluate the broader impact of workflow improvements achieved through mobile devices, including their effects on clinical outcomes, patient and caregiver satisfaction, cost reduction, and error minimization. Additionally, exploring effective strategies to promote this beneficial feature to patients and caregivers would help enhance awareness and utilization, supporting wider adoption of such systems.

Significant differences in usability were found according to age group. Demographic characteristics reportedly impact the usability of healthcare applications using mobile devices. 20 21 Similar to our results, older individuals exhibited lower usability and frequency of using mobile health applications than younger individuals in other studies, 22 23 highlighting the broader issue of mobile health applications. Despite providing admission process services through basic messaging applications and SMS, fewer older patients adopted the mobile services, indicating a challenge in the mobile health field. Additionally, consistent with our results, a previous study found higher mobile health application usage among female patients. 24 A higher usage frequency was also observed among patients undergoing surgery. Apart from potential associations with the non-surgery group's demographics, surgical patients may be more interested in their treatment and prefer a predictable treatment process.

### Limitations

This study has several limitations. First, as a retrospective study, selection bias due to differences in sample sizes between the groups is possible. However, with data from 7,000 individuals, this bias was minimized. Second, whether the patient or caregiver was the actual subject of the hospital admission process could not be determined, which may have affected the results. Children and adults with mobility difficulties require a legally responsible adult caregiver for admission. Although this bias may have been minimized, additional prospective studies are required for more accurate results. Third, data obtained from real-life situations may not accurately reflect the time required for the entire admission process due to factors such as bed congestion. To address this and minimize bias, median values and a sufficient sample size were used. Fourth, as this study was conducted at a single institution, the findings may not be generalizable to other healthcare settings, which could be addressed through multi-center studies. Fifth, confounding factors such as education level, income, and familiarity with mobile devices may have influenced the choice of mobile admission, although these effects were likely minimized due to the sample size. Sixth, the exclusion of a considerable amount of missing data may have introduced bias. However, as sufficient data were collected over a considerable period, missing data likely stemmed from inefficiencies in workflow compliance. The significance of this study lies in its potential to reduce such inefficiencies in the future. Lastly, the findings of this study have not been directly linked to clinical benefits for patients. Future qualitative research with a structured design, or comparative studies examining clinical outcomes between patients and caregivers who used mobile devices and those who did not, could further strengthen the significance and implications of the present findings.

## Conclusion

The mobile admission process can reduce task completion time compared with the traditional walk-in admission process.

## Clinical Relevance Statement

Implementing a mobile admission process significantly enhances hospital workflow efficiency by reducing admission turnaround time compared with traditional walk-in methods. This improvement allows healthcare institutions to optimize administrative operations, allocate resources more effectively, and enhance patient satisfaction, particularly among younger and tech-savvy populations. By addressing the needs of diverse demographics, including older patients needing additional support, the findings underscore the importance of integrating user-friendly technology into hospital admission systems. These insights can inform future digital health innovations to improve patient experiences and streamline hospital operations.

## Multiple-Choice Questions

1. What was the primary objective of this study?
  - To assess patient satisfaction with hospital care
  - To compare the time efficiency of mobile and walk-in admission processes
  - To evaluate the accuracy of electronic medical records
  - To analyze the financial benefits of telemedicine
  **Correct Answer** : The correct option is b. The primary outcome of this study was the time interval associated with two different admission processes.
2. How much time did the mobile admission process reduce compared with the traditional walk-in process?
  - 5.8 minutes
  - 8.8 minutes
  - 10.2 minutes
  - 12.6 minutes
  **Correct Answer** : The correct option is b. The mobile admission process was 8.8 minutes faster than the traditional walk-in process.
