# Supplementary material for: Mobile Admission Process and Administrative Turnaround Time for Hospitalization of Outpatients: A Retrospective Study
Source: Appl Clin Inform. 2025 Aug 14;16(4):769–76. doi: 10.1055/a-2576-7110 (PMC12352987; doi:10.1055/a-2576-7110)
Supplement: Supplementary file 1 — Supplementary Material [file 10-1055-a-2576-7110_26737717.pdf]

Supplementary Appendix 1 Score results ($n = 40$) of the System Usability Scale to assess the mobile admission process

No.	Statement	Mean (SD)
1	I think I will need to use the mobile admission process again.	4.3 (1.3)
2	The mobile admission process is not unnecessarily complicated.	1.4 (4.2)
3	The mobile admission process is easy to use.	4.2 (1.4)
4	Technical assistance is needed to use the mobile admission process.	1.9 (1.4)
5	The mobile admission process is well integrated with other features.	3.9 (1.2)
6	The mobile admission process lacks consistency.	1.8 (1.0)
7	Most people will quickly learn how to use the mobile admission process.	4.0 (1.2)
8	The mobile admission process is inconvenient to use.	1.5 (1.2)
9	I felt confident using the mobile admission process.	4.5 (1.0)
10	A lot of learning is required before using the mobile admission process.	1.7 (1.1)
	Total score	74.4 (14.5)

Abbreviation: SD, standard deviation

 © 2025. The Author(s).
